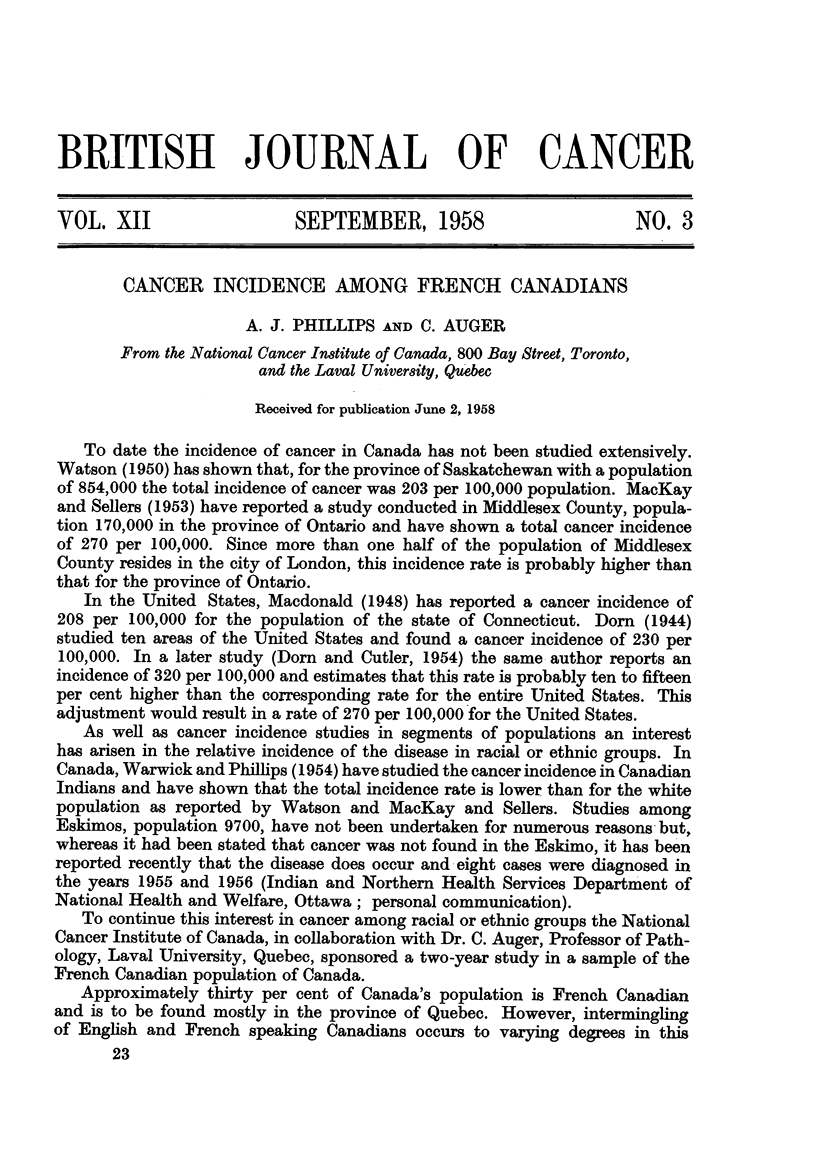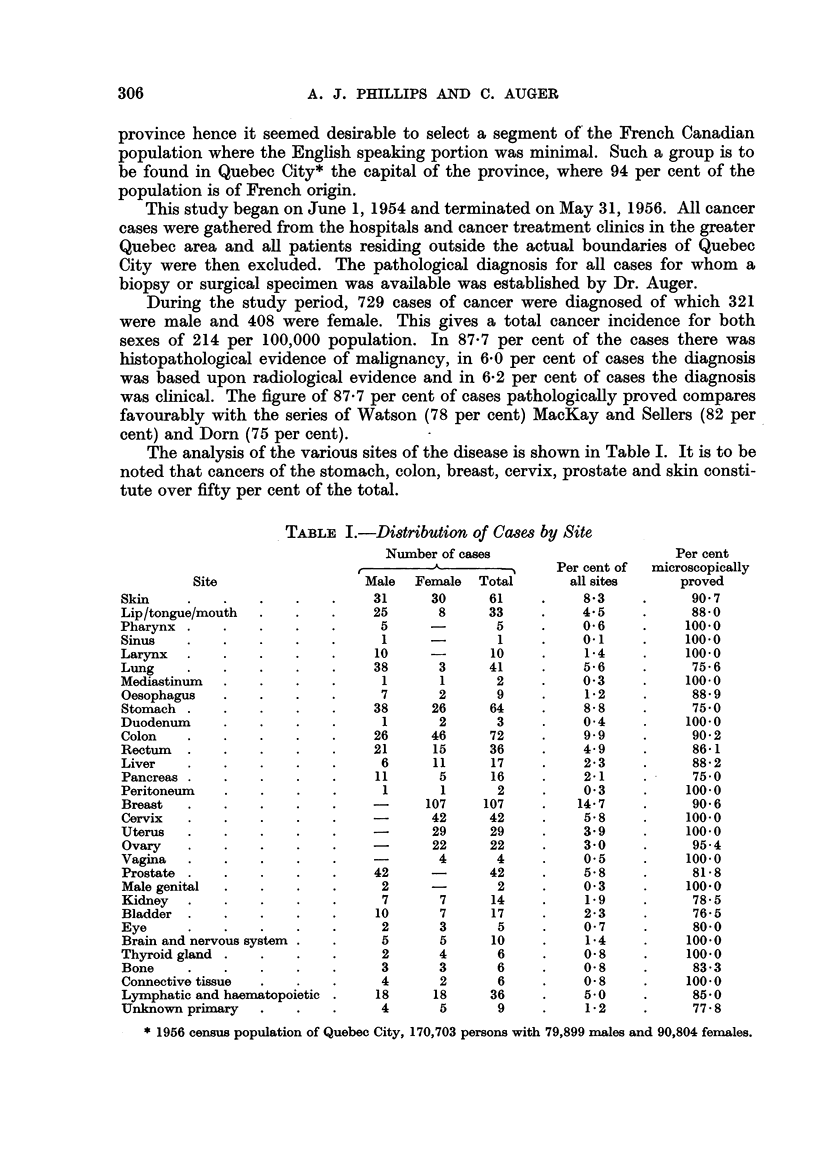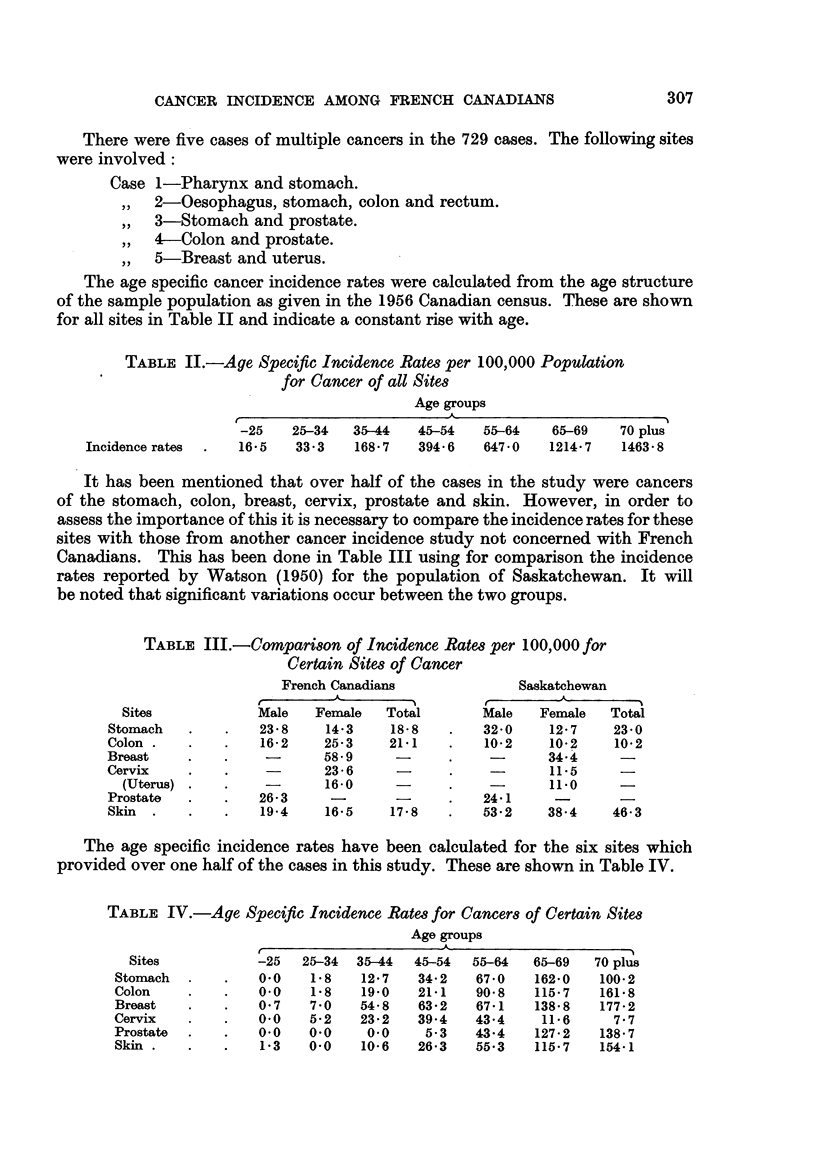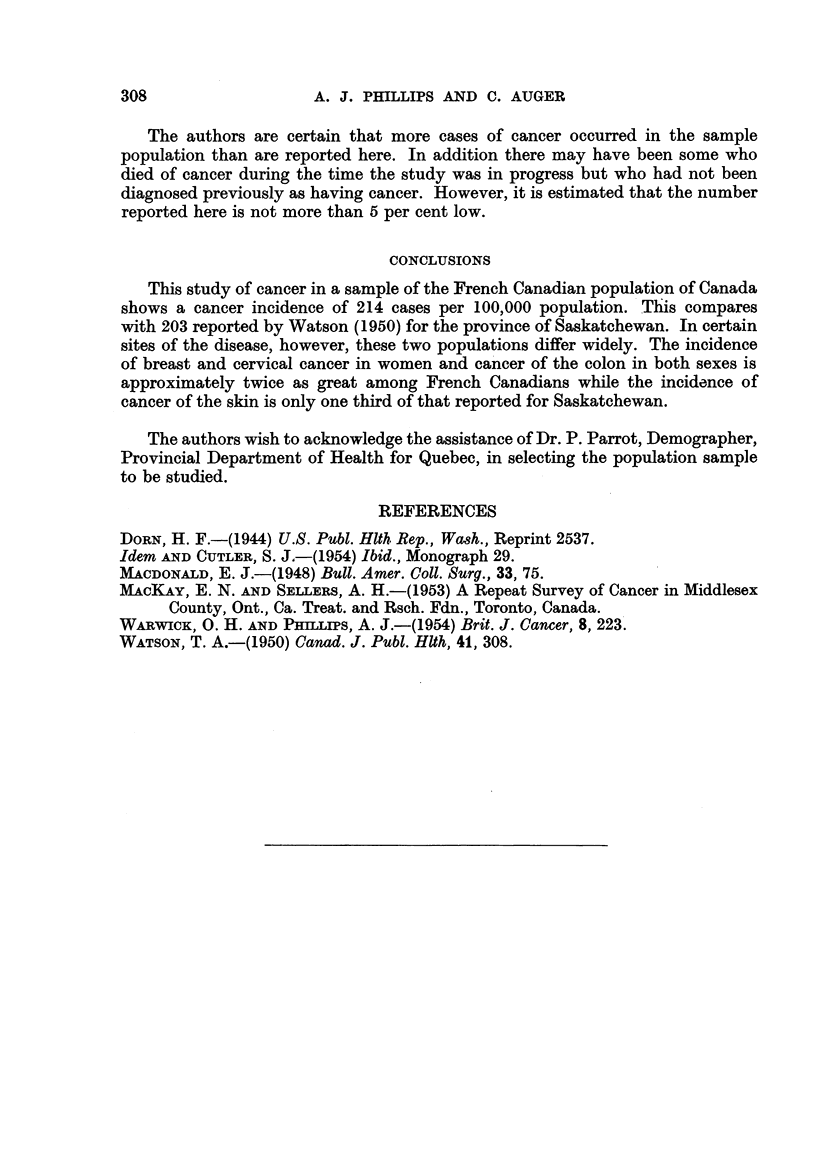# Cancer Incidence Among French Canadians

**DOI:** 10.1038/bjc.1958.36

**Published:** 1958-09

**Authors:** A. J. Phillips, C. Auger


### BRITISH JOURNAL OF CANCER

VOL. XII SEPTEMBER, 1958 NO. 3

### CANCER INCIDENCE AMONG FRENCH CANADIANS

A. J. PHILLIPS AND C. AUGER

*From the National Cancer Institute of Canada, 800 Bay Street, Toronto, and the Laval University, Quebec*

Received for publication June 2, 1958

To date the incidence of cancer in Canada has not been studied extensively. Watson (1950) has shown that, for the province of Saskatchewan with a population of 854,000 the total incidence of cancer was 203 per 100,000 population. MacKay and Sellers (1953) have reported a study conducted in Middlesex County, population 170,000 in the province of Ontario and have shown a total cancer incidence of 270 per 100,000. Since more than one half of the population of Middlesex County resides in the city of London, this incidence rate is probably higher than that for the province of Ontario.

In the United States, Macdonald (1948) has reported a cancer incidence of 208 per 100,000 for the population of the state of Connecticut. Dorn (1944) studied ten areas of the United States and found a cancer incidence of 230 per 100,000. In a later study (Dorn and Cutler, 1954) the same author reports an incidence of 320 per 100,000 and estimates that this rate is probably ten to fifteen per cent higher than the corresponding rate for the entire United States. This adjustment would result in a rate of 270 per 100,000 for the United States.

As well as cancer incidence studies in segments of populations an interest has arisen in the relative incidence of the disease in racial or ethnic groups. In Canada, Warwick and Phillips (1954) have studied the cancer incidence in Canadian Indians and have shown that the total incidence rate is lower than for the white population as reported by Watson and MacKay and Sellers. Studies among Eskimos, population 9700, have not been undertaken for numerous reasons but, whereas it had been stated that cancer was not found in the Eskimo, it has been reported recently that the disease does occur and eight cases were diagnosed in the years 1955 and 1956 (Indian and Northern Health Services Department of National Health and Welfare, Ottawa ; personal communication).

To continue this interest in cancer among racial or ethnic groups the National Cancer Institute of Canada, in collaboration with Dr. C. Auger, Professor of Pathology, Laval University, Quebec, sponsored a two-year study in a sample of the French Canadian population of Canada.

Approximately thirty per cent of Canada's population is French Canadian and is to be found mostly in the province of Quebec. However, intermingling of English and French speaking Canadians occurs to varying degrees in this province hence it seemed desirable to select a segment of the French Canadian population where the English speaking portion was minimal. Such a group is to be found in Quebec City* the capital of the province, where 94 per cent of the population is of French origin.

This study began on June 1, 1954 and terminated on May 31, 1956. All cancer cases were gathered from the hospitals and cancer treatment clinics in the greater Quebec area and all patients residing outside the actual boundaries of Quebec City were then excluded. The pathological diagnosis for all cases for whom a biopsy or surgical specimen was available was established by Dr. Auger.

During the study period, 729 cases of cancer were diagnosed of which 321 were male and 408 were female. This gives a total cancer incidence for both sexes of 214 per 100,000 population. In 87·7 per cent of the cases there was histopathological evidence of malignancy, in 6·0 per cent of cases the diagnosis was based upon radiological evidence and in 6·2 per cent of cases the diagnosis was clinical. The figure of 87·7 per cent of cases pathologically proved compares favourably with the series of Watson (78 per cent) MacKay and Sellers (82 per cent) and Dorn (75 per cent).

The analysis of the various sites of the disease is shown in Table I. It is to be noted that cancers of the stomach, colon, breast, cervix, prostate and skin constitute over fifty per cent of the total.

TABLE I.—*Distribution of Cases by Site*

| Site | Number of cases | | | Per cent of all sites | Per cent microscopically proved |
|---|---|---|---|---|---|
| | Male | Female | Total | | |
| Skin | 31 | 30 | 61 | 8·3 | 90·7 |
| Lip/tongue/mouth | 25 | 8 | 33 | 4·5 | 88·0 |
| Pharynx | 5 | — | 5 | 0·6 | 100·0 |
| Sinus | 1 | — | 1 | 0·1 | 100·0 |
| Larynx | 10 | — | 10 | 1·4 | 100·0 |
| Lung | 38 | 3 | 41 | 5·6 | 75·6 |
| Mediastinum | 1 | 1 | 2 | 0·3 | 100·0 |
| Oesophagus | 7 | 2 | 9 | 1·2 | 88·9 |
| Stomach | 38 | 26 | 64 | 8·8 | 75·0 |
| Duodenum | 1 | 2 | 3 | 0·4 | 100·0 |
| Colon | 26 | 46 | 72 | 9·9 | 90·2 |
| Rectum | 21 | 15 | 36 | 4·9 | 86·1 |
| Liver | 6 | 11 | 17 | 2·3 | 88·2 |
| Pancreas | 11 | 5 | 16 | 2·1 | 75·0 |
| Peritoneum | 1 | 1 | 2 | 0·3 | 100·0 |
| Breast | — | 107 | 107 | 14·7 | 90·6 |
| Cervix | — | 42 | 42 | 5·8 | 100·0 |
| Uterus | — | 29 | 29 | 3·9 | 100·0 |
| Ovary | — | 22 | 22 | 3·0 | 95·4 |
| Vagina | — | 4 | 4 | 0·5 | 100·0 |
| Prostate | 42 | — | 42 | 5·8 | 81·8 |
| Male genital | 2 | — | 2 | 0·3 | 100·0 |
| Kidney | 7 | 7 | 14 | 1·9 | 78·5 |
| Bladder | 10 | 7 | 17 | 2·3 | 76·5 |
| Eye | 2 | 3 | 5 | 0·7 | 80·0 |
| Brain and nervous system | 5 | 5 | 10 | 1·4 | 100·0 |
| Thyroid gland | 2 | 4 | 6 | 0·8 | 100·0 |
| Bone | 3 | 3 | 6 | 0·8 | 83·3 |
| Connective tissue | 4 | 2 | 6 | 0·8 | 100·0 |
| Lymphatic and haematopoietic | 18 | 18 | 36 | 5·0 | 85·0 |
| Unknown primary | 4 | 5 | 9 | 1·2 | 77·8 |

* 1956 census population of Quebec City, 170,703 persons with 79,899 males and 90,804 females.

There were five cases of multiple cancers in the 729 cases. The following sites were involved :

Case 1—Pharynx and stomach.
,, 2—Oesophagus, stomach, colon and rectum.
,, 3—Stomach and prostate.
,, 4—Colon and prostate.
,, 5—Breast and uterus.

The age specific cancer incidence rates were calculated from the age structure of the sample population as given in the 1956 Canadian census. These are shown for all sites in Table II and indicate a constant rise with age.

TABLE II.—*Age Specific Incidence Rates per* 100,000 *Population for Cancer of all Sites*

| | Age groups | | | | | | |
|---|---|---|---|---|---|---|---|
| | -25 | 25–34 | 35–44 | 45–54 | 55–64 | 65–69 | 70 plus |
| Incidence rates . | 16·5 | 33·3 | 168·7 | 394·6 | 647·0 | 1214·7 | 1463·8 |

It has been mentioned that over half of the cases in the study were cancers of the stomach, colon, breast, cervix, prostate and skin. However, in order to assess the importance of this it is necessary to compare the incidence rates for these sites with those from another cancer incidence study not concerned with French Canadians. This has been done in Table III using for comparison the incidence rates reported by Watson (1950) for the population of Saskatchewan. It will be noted that significant variations occur between the two groups.

TABLE III.—*Comparison of Incidence Rates per* 100,000 *for Certain Sites of Cancer*

| | French Canadians | | | Saskatchewan | | |
|---|---|---|---|---|---|---|
| Sites | Male | Female | Total | Male | Female | Total |
| Stomach . . | 23·8 | 14·3 | 18·8 | 32·0 | 12·7 | 23·0 |
| Colon . . . | 16·2 | 25·3 | 21·1 | 10·2 | 10·2 | 10·2 |
| Breast . . | — | 58·9 | — | — | 34·4 | — |
| Cervix . . | — | 23·6 | — | — | 11·5 | — |
| (Uterus) . . | — | 16·0 | — | — | 11·0 | — |
| Prostate . . | 26·3 | — | — | 24·1 | — | — |
| Skin . . . | 19·4 | 16·5 | 17·8 | 53·2 | 38·4 | 46·3 |

The age specific incidence rates have been calculated for the six sites which provided over one half of the cases in this study. These are shown in Table IV.

TABLE IV.—*Age Specific Incidence Rates for Cancers of Certain Sites*

| | Age groups | | | | | | |
|---|---|---|---|---|---|---|---|
| Sites | -25 | 25–34 | 35–44 | 45–54 | 55–64 | 65–69 | 70 plus |
| Stomach . . | 0·0 | 1·8 | 12·7 | 34·2 | 67·0 | 162·0 | 100·2 |
| Colon . . | 0·0 | 1·8 | 19·0 | 21·1 | 90·8 | 115·7 | 161·8 |
| Breast . . | 0·7 | 7·0 | 54·8 | 63·2 | 67·1 | 138·8 | 177·2 |
| Cervix . . | 0·0 | 5·2 | 23·2 | 39·4 | 43·4 | 11·6 | 7·7 |
| Prostate . . | 0·0 | 0·0 | 0·0 | 5·3 | 43·4 | 127·2 | 138·7 |
| Skin . . . | 1·3 | 0·0 | 10·6 | 26·3 | 55·3 | 115·7 | 154·1 |

The authors are certain that more cases of cancer occurred in the sample population than are reported here. In addition there may have been some who died of cancer during the time the study was in progress but who had not been diagnosed previously as having cancer. However, it is estimated that the number reported here is not more than 5 per cent low.

### CONCLUSIONS

This study of cancer in a sample of the French Canadian population of Canada shows a cancer incidence of 214 cases per 100,000 population. This compares with 203 reported by Watson (1950) for the province of Saskatchewan. In certain sites of the disease, however, these two populations differ widely. The incidence of breast and cervical cancer in women and cancer of the colon in both sexes is approximately twice as great among French Canadians while the incidence of cancer of the skin is only one third of that reported for Saskatchewan.

The authors wish to acknowledge the assistance of Dr. P. Parrot, Demographer, Provincial Department of Health for Quebec, in selecting the population sample to be studied.

### REFERENCES

DORN, H. F.—(1944) *U.S. Publ. Hlth Rep., Wash.*, Reprint 2537.
*Idem* AND CUTLER, S. J.—(1954) *Ibid.*, Monograph 29.
MACDONALD, E. J.—(1948) *Bull. Amer. Coll. Surg.*, **33**, 75.
MACKAY, E. N. AND SELLERS, A. H.—(1953) A Repeat Survey of Cancer in Middlesex County, Ont., Ca. Treat. and Rsch. Fdn., Toronto, Canada.
WARWICK, O. H. AND PHILLIPS, A. J.—(1954) *Brit. J. Cancer*, **8**, 223.
WATSON, T. A.—(1950) *Canad. J. Publ. Hlth*, **41**, 308.